# Transcriptome Dynamics Reveal the Potential Roles of Long Non-Coding RNAs in Regulating Flower Color of Safflowers (*Carthamus tinctorius*)

**DOI:** 10.3390/ijms27115142

**Published:** 2026-06-05

**Authors:** Saimire Aishan, Shuo Liu, Lu Lv, Jian Wei, Zhaojun Wei, Jiao Liu, Hong Liu, Rui Qin

**Affiliations:** 1 Hubei Provincial Key Laboratory for Protection and Application of Special Plant Germplasm in Wuling Area of China, College of Life Sciences, South-Central Minzu University, Wuhan 430074, China; samira_hs@163.com (S.A.); liushuoyl@163.com (S.L.); lvlu@mail.scuec.edu.cn (L.L.); jiao.liu@scuec.edu.cn (J.L.); 2 Institute for Safflower Industry Research, Shihezi University, Shihezi 832003, China; lan18946612103@shzu.edu.cn; 3 School of Biological Science and Engineering, North Minzu University, Yinchuan 750021, China

**Keywords:** *Carthamus tinctorius*, *CtCHS.7*, flower color, flavonoid biosynthesis, long non-coding RNA, prokaryotic expression, RNA sequencing

## Abstract

Safflower (*Carthamus tinctorius* L.) is an important medicinal plant widely used as a source of natural pigments. Flower color is a key trait affecting both ornamental and commercial value; however, the roles of long non-coding RNAs (lncRNAs) in safflower flower coloration remain largely unclear. In this study, strand-specific RNA sequencing was performed on three safflower varieties with distinct flower colors at different floral developmental stages. A total of 4851 lncRNAs were identified, including 940 natural antisense transcript (NAT) pairs. Among them, lncRNA *MSTRG.28365* was identified as a natural antisense transcript paired with *CtCHS.7*, a chalcone synthase-like gene potentially involved in flavonoid biosynthesis. Expression analysis revealed that *CtCHS.7* was highly expressed in the red-flowered variety, whereas MSTRG.28365 exhibited an opposite expression pattern, suggesting a potential regulatory association. Co-expression analysis further indicated that *CtCHS.7* was associated with genes putatively involved in flavonoid modification, including UDP-glycosyltransferases and cytochrome P450 enzymes. Functional assays showed that the recombinant *CtCHS.7* protein could catalyze the production of downstream flavonoid-related metabolites, although the detected product differed from canonical naringenin chalcone. These findings suggest that lncRNAs may participate in flower color variation and flavonoid biosynthesis-related processes in safflower. This study provides candidate regulatory elements for future functional validation of safflower flower coloration mechanisms.

## 1. Introduction

Safflower (*Carthamus tinctorius* L.) is a plant of the genus *Carthamus* in the family Asteraceae. It is generally believed to have originated in the Near East or eastern Mediterranean region, although its precise domestication history remains controversial [1,2]. The crop was later introduced into Egypt, India, Central Asia, and East Asia through ancient cultivation and trade activities, resulting in abundant genetic diversity across different geographic regions. Safflower has been cultivated in China for over two millennia and is widely used as a medicinal herb, oilseed crop, animal feed, and source of natural dyes and food colorants [3,4]. Modern pharmacological studies have confirmed that safflower exhibits extensive pharmacological effects in the cardiovascular and cerebrovascular systems, hematological system, and nervous system, including antioxidant, neuroprotective, and anti-inflammatory activities [5]. Safflower cultivars display diverse flower color phenotypes, ranging from yellow and orange to red and white, with a significant positive correlation between pigment accumulation levels and flower color intensity [6].

Flower color is a key trait determining both ornamental value and horticultural applications [7]. It is primarily governed by the biosynthesis and accumulation of pigments such as flavonoids, carotenoids, and betalains [7]. Among these, anthocyanins primarily contribute to red, purple, and blue coloration, while flavones and flavonols act as co-pigments that stabilize anthocyanin-based coloration [8]. For example, whole-transcriptome analysis in *Chrysanthemum seticuspe* revealed complex mRNA–miRNA–lncRNA regulatory networks associated with ray and disk floret development, suggesting that lncRNAs may participate in the regulation of floral traits and pigment-related metabolic processes [9]. In lotus, flower color diversity is associated with differential expression of key flavonoid biosynthetic genes, including chalcone isomerase, dihydroflavonol 4-reductase, and anthocyanidin synthase, which regulate flavonoid biosynthesis and contribute to diverse floral phenotypes [10].

Long non-coding RNAs (lncRNAs), including long intergenic non-coding RNAs (lincRNAs) and natural antisense transcripts (lncNATs), are important regulatory components of eukaryotic genomes [11]. lncNATs are transcribed from the opposite strand of protein-coding genes within the same genomic regions and can regulate the expression of their corresponding sense transcripts through diverse mechanisms, such as chromatin modification, alternative splicing, miRNA regulation, and control of mRNA translation and stability [12,13]. An lncNAT and its paired sense transcript together constitute a natural antisense transcript (NAT) pair. Based on the relative orientation and genomic arrangement of sense and antisense transcripts, NAT pairs can be categorized into three types: divergent (5′-end overlap), convergent (3′-end overlap), and enclosed (one transcript fully embedded within the other). However, the characteristics and regulatory roles of NAT pairs in safflower remain largely unexplored.

As key regulatory components of eukaryotic genomes, long non-coding RNAs (lncRNAs) participate in diverse biological processes, including transcriptional regulation, chromatin remodeling, and secondary metabolism [14,15]. These lncRNAs have been shown to regulate genes through chromatDin structure modification, control of alternative splicing, fine-tuning of miRNA activity, and regulation of mRNA translation and accumulation [16,17]. For example, lncRNA1 identified in Salvia miltiorrhiza has been confirmed to function as a competitive endogenous RNA that sequesters miR156a, thereby relieving the repression of the target gene *SmSPL12* and ultimately activating the expression of key enzyme genes in the flavonoid biosynthesis pathway, promoting danshensu accumulation. Another study in apples revealed that *MLNC3.2* and *MLNC4.6* can interact with the *MdMYB1* transcription factor, enhancing its binding affinity to the promoters of the DFR and UFGT genes and positively regulating anthocyanin biosynthesis. Moreover, numerous studies have demonstrated that lncRNAs play important roles in regulating flower color in plants [18,19]. However, research on the role of lncRNAs in safflower flower color formation remains relatively limited.

Natural antisense transcripts (NATs), particularly lncNATs, are increasingly recognized as important regulators of plant secondary metabolism. Recent studies have demonstrated that NATs regulate biosynthetic genes through transcriptional interference, RNA duplex formation, chromatin remodeling, and interactions with small RNAs, thereby influencing specialized metabolite accumulation in medicinal plants [14,20]. Because flavonoids and quinochalcones are the major pigment-related secondary metabolites in safflower petals, lncNAT-mediated regulation may contribute to the transcriptional control of flavonoid biosynthesis and flower color formation in safflower. However, research on the role of lncRNAs in safflower flower color formation remains relatively limited.

In this study, safflower was used as the experimental material, and strand-specific RNA sequencing (ssRNA-seq) was conducted on flowers of three color variants (red, yellow, and white) at both the bud and flowering stages. A total of 940 coding–noncoding natural antisense transcript (NAT) pairs were identified, of which 249 were identified as candidate NAT pairs potentially involved in coding gene expression. Notably, chalcone synthase (CHS), the rate-limiting enzyme in flavonoid biosynthesis, as well as its downstream modification genes, were found to be regulated by lncRNAs. These findings reveal numerous lncNATs involved in safflower flower color formation, providing novel insights into the molecular mechanisms of flower pigmentation and valuable targets for future genetic improvement.

## 2. Results

### 2.1. Flower Color Variation and Long Non-Coding RNAs Identification

**Figure 1:**
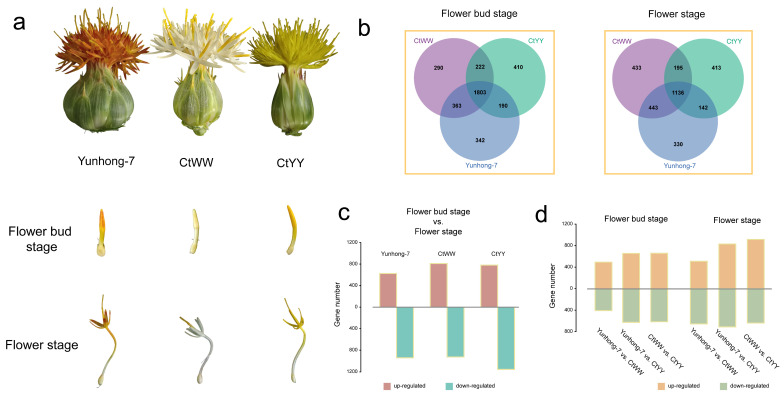
Identification and differential expression analysis of long non-coding RNAs (lncRNAs) in safflower. (**a**). Growth phenotypes of three safflower varieties with different flower colors at the flower bud stage and flowering stage. (**b**). Venn graph of lncRNAs of three safflower varieties in the flower bud stage and flowering stage. (**c**). The number of differentially expression lncRNAs between flower bud stage and flowering stage. (**d**). The number of differentially expression lncRNAs across three safflower varieties.

The flower of safflower (*Carthamus tinctorius*) is composed of numerous tubular florets aggregated together. Significant variation in flower color exists within the safflower population, with three predominant types: red, yellow, and white flowers. This variation is primarily attributed to differences in anthocyanin accumulation, a major class of flavonoid compounds synthesized through the flavonoid biosynthesis pathway. To investigate the role of long non-coding RNAs (lncRNAs) in flower color regulation in safflower, strand-specific transcriptome sequencing was performed on tubular florets of three safflower varieties (Yunhong-7, CtWW, and CtYY) with distinct flower colors at both the flower bud stage and the flowering stage (Figure 1a).

Yunhong-7 exhibits a red final flower color, whereas CtWW and CtYY display white and yellow flowers, respectively. A total of 433.4 million high-quality paired-end reads (2 × 150 bp) were retained across the two developmental stages for the three safflower varieties (Supplementary Table S2). These reads were subsequently aligned to the Yunhong-7 reference genome, and transcript assembly was performed for each sample based on the alignment results. The assembled transcripts were then merged across all samples to generate a transcriptome set containing 20,725 genes and 46,108 transcripts.

To validate the reliability of the transcriptome sequencing (RNA-seq) results, four candidate genes were selected for qPCR analysis. The qPCR results showed expression patterns consistent with the RNA-seq data (Supplementary Figure S1), confirming the reliability of the transcriptome results. These four genes were selected because they showed representative differential expression patterns across varieties and developmental stages and covered a range of expression levels in the RNA-seq dataset.

A total of 4851 lncRNAs were identified in safflower, with 3620 and 3092 expressed at the flower bud and flowering stages, respectively (Supplementary Tables S2 and S3). Among them, 1803 (49.8%) were shared across the three safflower varieties with distinct flower colors at the flower bud stage, whereas only 1136 (36.7%) were commonly expressed at the flowering stage (Figure 1b; Supplementary Table S4). Pearson correlation analysis of transcript expression revealed that mRNAs exhibited higher expression stability than lncRNAs (Supplementary Figures S2 and S3). The number of expressed lncRNAs was markedly lower at the flowering stage than at the flower bud stage, and the proportion shared among all three varieties was further reduced, indicating increased variety-specific expression.

The differentially expressed transcripts suggest a potential association with genetic variation among safflower varieties or with regulatory divergence across developmental stages. A total of 3093 differentially expressed lncRNAs were identified between flower bud stage and flowering stage, including 1564 in Yunhong-7, 1733 in CtWW, and 1935 in CtYY (Figure 1c, Supplementary Table S5). The number of lncRNAs down-regulated during the flowering stage was significantly greater than those up-regulated. In addition, 1947 and 2325 differentially expressed lncRNAs were identified across three safflower varieties at the flower bud stage and flowering stage, respectively (Figure 1d). Given that the flowering stage exhibits fewer total expressed lncRNAs, yet displays a higher number of differentially expressed lncRNAs compared to the flower bud stage, this further supports the conclusion that lncRNAs tend to exhibit variety-specific expression during the flowering stage.

### 2.2. Identification of Natural Antisense Transcript Pairs (NAT)

**Figure 2:**
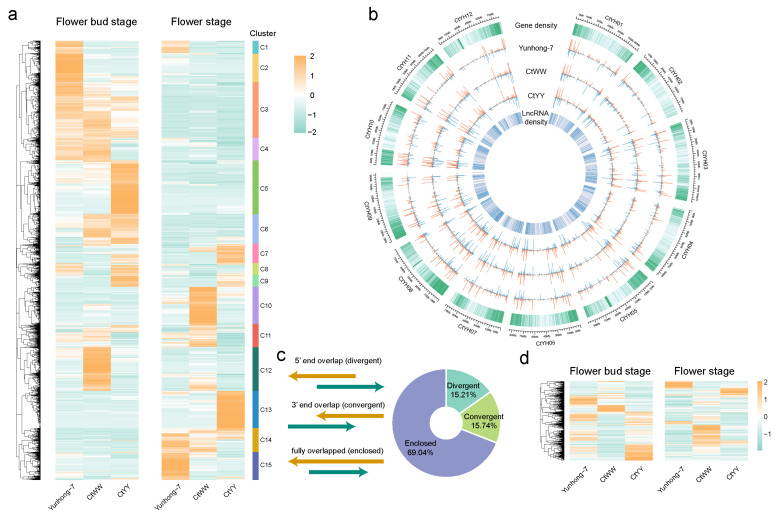
Genome wide identification of natural antisense transcript (NAT) pairs. (**a**). Expression profiles of lncRNAs in three safflower varieties (Yunhong-7, CtWW, CtYY). (**b**). Distribution patterns of lncRNAs in the genome chromosome. The orange-yellow and blue lines represent the expression levels of up-regulated and down-regulated lncRNAs, respectively. (**c**). Classification of NAT pairs based on the direction of transcription and the overlap region between sense and antisense transcripts. Orange arrows represent sense transcripts, while cyan arrows represent antisense transcripts. (**d**). Expression profiles of lncNATs in three safflower varieties.

The heatmap of 3876 differentially expressed lncRNAs clearly reveals both tissue-specific and variety-specific expression patterns of lncRNAs (Figure 2a). For instance, gene cluster C1 was differentially upregulated in Yunhong-7, C3 at the flower bud stage across all safflower varieties, and C13 exclusively during the flowering stage in CtYY (Figure 2a). Subsequently, comparison of the genomic distributions of lncRNAs and protein-coding genes revealed a high degree of consistency in their density patterns. Moreover, lncRNAs located in genomic regions with higher gene density tend to exhibit higher expression levels (Figure 2b). Over the past two decades, research has revealed that lncRNAs modulate transcriptional programs by directly interacting with the transcriptional machinery to repress or activate it, and they have also been shown to play critical regulatory roles in pre-mRNA splicing [21]. Therefore, the clustered distribution and elevated expression of lncRNAs in genomic regions with high gene density suggest their potential role in regulating protein-coding genes in safflower.

To investigate the regulatory role of lncRNAs in modulating coding genes in safflower, a total of 940 natural antisense transcript (NAT) pairs were identified based on the genomic positions of lncRNAs relative to protein-coding genes. Based on the relative orientation and genomic arrangement of sense and antisense transcripts, these NAT pairs were classified into three categories: divergent, convergent, and enclosed. Among them, 15.21% were divergent, 15.74% were convergent, and 69.04% were enclosed, indicating that the enclosed type represents the predominant configuration (Figure 2c).

At the genome-wide level, mRNAs exhibited higher expression levels compared to lncRNAs and lncNATs during both the flower bud and flowering stages (Supplementary Figure S4). Further analysis of lncNAT expression revealed that, during the flowering stage in safflower, lncNATs exhibit more pronounced variety-specific expression compared to the flower bud stage (Figure 2d). Therefore, GO enrichment results revealed that lncNATs were involved in multiple important biological processes, including “defense response”, “transmembrane transport”, “DNA repair” and “intracellular signal transduction” (Supplementary Figure S5).

### 2.3. lncNATs Potentially Associated with Flower Color-Related Genes

**Figure 3:**
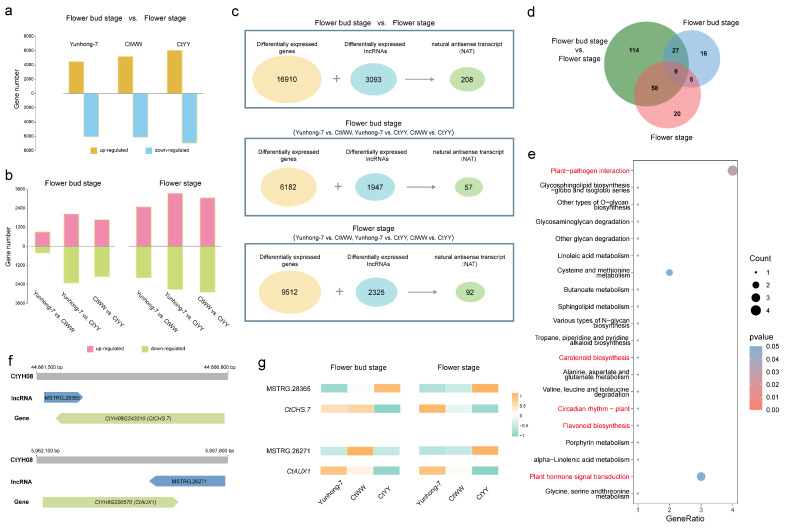
Analysis of coding genes and NAT pairs. (**a**). The number of differentially expression genes (DEGs) between flower bud stage and flowering stage. (**b**). The number of DEGs across three safflower varieties. (**c**). Identification of NAT pairs in which lncNATs exhibit potential regulatory associations with protein-coding genes. (**d**). Venn graph of lncNATs which exhibit regulatory relationships with protein-coding genes of three comparative groups. (**e**). Kyoto Encyclopedia of Genes and Genomes (KEGG) enrichment analysis of lncNATs which exhibit regulatory relationships with protein-coding genes. Red-highlighted pathways are related to flower color, defense responses, and flowering regulation. (**f**). Visualization of the genomic positions of lncNATs and protein-coding genes within key NAT pairs (*CtCHS.7* and MSTRG.28365, *CtAUX1* and MSTRG.26271). (**g**). Expression profiles of two key transcript within key NAT pairs (*CtCHS.7* and MSTRG.28365, *CtAUX1* and MSTRG.26271).

A total of 16,910 differentially expressed genes (DEGs) were identified between flower bud stage and flowering stage, including 10,545 in Yunhong-7, 11,312 in CtWW, and 13,002 in CtYY (Figure 3a, Supplementary Table S6). Similarly, a greater number of genes exhibited down-regulated expression during the flowering stage. Meanwhile, 6182 and 9512 DEGs were identified across three safflower varieties at the flower bud stage and flowering stage, respectively (Figure 3b). Notably, Yunhong-7 and CtWW exhibited the fewest DEGs at the flower bud stage, with only 1314 DEGs identified, suggesting a high degree of transcriptional similarity between these two safflower varieties during this developmental phase (Figure 3b).

The number of DEGs was substantially higher than that of differentially expressed lncRNAs. This observation suggests that only a subset of transcriptional variation may be associated with lncRNA-related regulation. Therefore, we further identified lncNATs potentially involved in the regulation of coding genes. A lncNAT–coding gene pair was considered a candidate regulatory association when both transcripts were differentially expressed and formed a NAT pair. A total of 208 NAT pairs exhibiting potential regulatory associations were identified between the flower bud stage and the flowering stage. In addition, 57 and 92 such NAT pairs were respectively detected among the three safflower varieties at the flower bud stage and the flowering stage (Figure 3c, Supplementary Tables S6 and S7, Supplementary Figures S6 and S7). Among them, nine lncNATs are involved in differential regulation both between different floral developmental stages and among safflower varieties at the flower bud and flowering stages (Figure 3d). Further KEGG functional enrichment analysis revealed that lncNATs are involved in the regulation of multiple traits, including disease resistance, flower color, and flowering time in safflower. Enriched KEGG pathways include “plant–pathogen interaction”, “carotenoid biosynthesis”, “circadian rhythm-plant”, “flavonoid biosynthesis”, and “plant hormone signal transduction” (Figure 3e, Supplementary Table S8). Carotenoids and flavonoids are the decisive pigments determining flower color in many plant species [22,23]. Genes associated with the circadian rhythm are involved in the regulation of flowering time [24], and plant hormones regulate multiple traits, including flower development, flower color, and flowering time [25,26,27].

Chalcone synthase (CHS) is a key enzyme that catalyzes the first committed step in the flavonoid biosynthetic pathway. The analysis of NAT pairs reveals that *CtCHS.7* and MSTRG.28365 form a convergent-type NAT pair, as do *CtAUX1* and MSTRG.26271 (Figure 3f). Previous studies have suggested that some lncNATs regulate coding gene expression through mechanisms involving attenuation of RNA polymerase II elongation and premature transcription termination [28]. Expression analysis indicates that the expression pattern of MSTRG.28365 is inversely correlated to that of *CtCHS.7*, indicating a potential negative correlation between MSTRG.28365 and *CtCHS.7* expression (Figure 3g). The type and content of anthocyanins are key determinants influencing flower color formation, and the expression of the CHS gene provides essential substrates for anthocyanin accumulation [29]. *CtCHS.7* was differentially upregulated during the flowering stage in the safflower variety Yunhong-7, which exhibits red flowers, whereas CtWW and CtYY display white and yellow flowers, respectively, suggesting that elevated expression of *CtCHS.7* is associated with the red flower phenotype in Yunhong-7 (Figure 3g). The results support a potential association between lncNAT expression patterns and flavonoid biosynthesis-related genes involved in safflower flower coloration.

### 2.4. Gene Co-Expression Network Construction and Analysis

**Figure 4:**
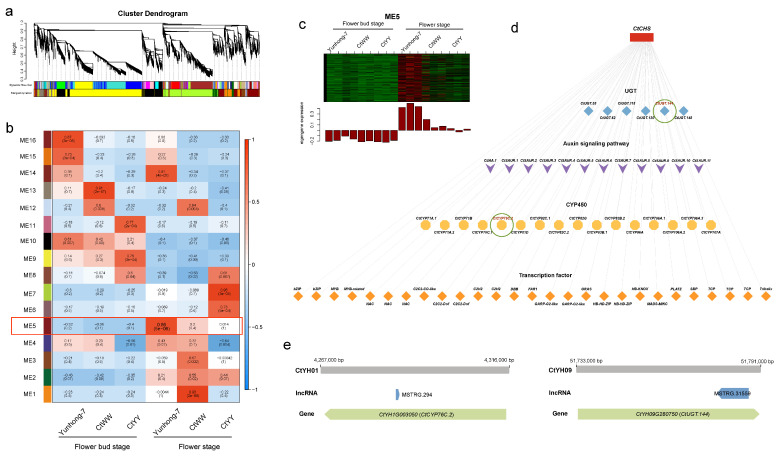
Weighted gene co-expression network analysis (WGCNA). (**a**). Gene cluster dendrograms and module detecting. (**b**). Heat map of module–trait correlation. The red box marks the module where *CtCHS.7* is located. (**c**). Expression pattern of module ME5. (**d**). Gene co-expression network analysis of *CtCHS.7* and other genes related to flavonoid biosynthesis. Red font and green circlesdenote UGT and CYP450 genes potentially affected by IncRNAs. (**e**). Visualization of the genomic positions of lncNATs and protein-coding genes within key NAT pairs (*CtCYP76C.2* and MSTRG.294, *CtUGT.144* and MSTRG.31559).

To identify potential downstream genes of CHS involved in flavonoid biosynthesis, a weighted gene co-expression network was constructed from the expression profiles of protein-coding genes using the WGCNA package (Figure 4a). A total of 16 modules were ultimately identified and designated as ME1 through ME16 (Figure 4b). The association between gene modules and the flower color trait was assessed, with six modules showing high correlation. ME5 (r = 0.88, *p* = 5 × 10^−8^) and ME16 (r = 0.87, *p* = 3 × 10^−6^) were significantly upregulated at the flowering stage and flower bud stage in Yunhong-7, respectively; ME1 (r = 0.93, *p* = 2 × 10^−8^) and ME13 (r = 0.91, *p* = 2 × 10^−7^) at the corresponding stages in CtWW; and ME7 (r = 0.95, *p* = 3 × 10^−9^) and ME11 (r = 0.77, *p* = 2 × 10^−4^) in CtYY (Figure 4b). The expression pattern of CtCHS.7 is consistent with that of the ME5 module, and therefore it is assigned to the ME5 module (Figure 4c). In the ME5 module, a total of 6 UDP-glycosyltransferase (UGT) genes, 12 auxin-related genes, 16 cytochrome P450 (P450) genes, and 27 transcription factors (TFs) are co-expressed with CtCHS.7 (Figure 4d). Among the transcription factors co-expressed with *CtCHS.7*, two belong to the basic leucine zipper (bZIP) family, two to the MYB family, and three to the NAC family. Previous studies have demonstrated that these transcription factor families are involved in the regulation of anthocyanin biosynthesis [30,31,32], suggesting potential involvement in flower color-associated regulatory networks.

In plants, flavonoids exist in various modified forms, and their structural diversity is primarily attributed to chemical modifications such as hydroxylation, methylation, acylation, and glycosylation, with glycosylated flavonoids representing the most prevalent natural form [33]. The CYP450 and UGT families respectively participate in catalyzing the hydroxylation and glycosylation reactions of flavonoids. Among the CYP450 and UGT genes co-expressed with *CtCHS.7*, *CtCYP76C.2* and *CtUGT.144* are also regulated by lncNATs. Specifically, *CtCYP76C.2* and MSTRG.294 form an enclosed-type NAT pair, and *CtUGT.144* and MSTRG.31559 likewise form an enclosed-type NAT pair (Figure 4e). These results suggest that lncNATs may participate not only in regulating the rate-limiting enzyme CHS, but also in modulating downstream flavonoid modification enzymes such as CYP450s and UGTs.

### 2.5. Functional Analysis of Candidate Flavonoid Biosynthesis Genes Linked to lncNATs

**Figure 5:**
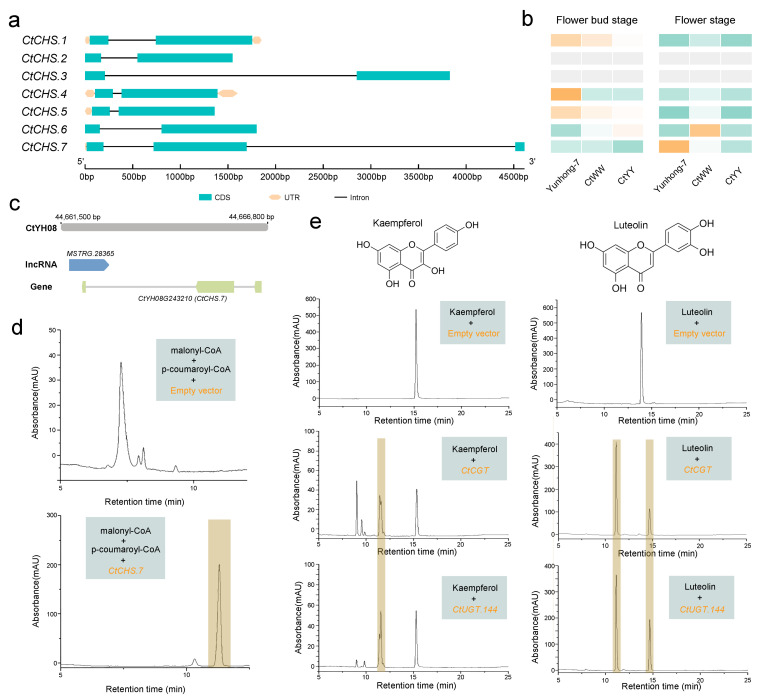
Analysis and functional validation of the CHS gene family in safflowers. (**a**). Analysis of the gene structure of the CHS gene family in safflowers. (**b**). Expression profiles of CHS genes in the flower bud stage and flowering stage. (**c**). Visualization of the position of lncNAT *MSTRG.28365* relative to *CtCHS.7* gene. (**d**). Prokaryotic expression of *CtCHS.7.* Using malonyl-CoA and p-coumaroyl-CoA as substrate. The bottom panel displays the chromatogram of the naringenin chalcone standard. (**e**). Prokaryotic expression of *CtUGT.144* and *CtCGT* using kaempferol and luteolin as substrate.

A total of seven CHS genes were identified in the safflower genome. With the exception of *CtCHS.7*, which contains three exons, the remaining six CHS genes each contain two exons, and the intron lengths among these seven genes exhibit considerable variation (Figure 5a). *CtCHS.2* and *CtCHS.3* were not expressed at either the flower bud stage or the flowering stage. *CtCHS.1* and *CtCHS.5* exhibited significant stage-specific expression patterns, showing high expression levels during the flower bud stage. In contrast, *CtCHS.4*, *CtCHS.6*, and *CtCHS.7* displayed variety-specific expression profiles: *CtCHS.4* was significantly upregulated exclusively at the flower bud stage in the Yunhong-7 variety; *CtCHS.6* was significantly upregulated solely at the flowering stage in the CtWW variety; and *CtCHS.7* was significantly upregulated specifically at the flowering stage in the Yunhong-7 variety (Figure 5b). Gene structure analysis indicates that *CtCHS.7* has an additional exon sequence at the 5′ end compared to other CHS genes in the safflower. Further analysis of the binding site between lncNATs *MSTRG.28365* and *CtCHS.7* reveals that the binding position precisely lies on this newly added exon sequence (Figure 5c). Therefore, it is hypothesized that during the evolution of safflower, *CtCHS.7* acquired an additional exon at the 5′ end via mutation events, which may have contributed to potential functional divergence from other CHS genes, thereby establishing it as a specific regulatory target of lncNATs.

In the flavonoid biosynthetic pathway, chalcone synthase encoded by the CHS gene catalyzes the condensation of malonyl-CoA and p-coumaroyl-CoA to form naringenin chalcone. The prokaryotic expression system is a widely used approach for validating gene functions [34,35]. To validate the function of *CtCHS.7* in safflower, we first purified the encoded enzyme protein and employed a prokaryotic expression system to assess its catalytic activity toward malonyl-CoA and p-coumaroyl-CoA. The results demonstrated that, compared with the empty vector control, the CtCHS.7 encoded enzyme was capable of catalyzing the formation of new metabolites, indicating that the *CtCHS.7* enzyme can produce downstream compounds in the flavonoid biosynthetic pathway (Figure 5d, Supplementary Figure S7). Further comparison of the peak time of the new metabolite produced by the *CtCHS.7* encoded enzyme with that of naringenin chalcone revealed that they were not consistent, suggesting that the product may differ from canonical naringenin chalcone. This observation raises the possibility that CtCHS.7 may possess catalytic properties distinct from canonical CHS enzymes, potentially associated with its distinct exon structure.

The enzyme encoded by *CtCGT* is the key enzyme responsible for hydroxysafflor yellow A (HSYA) biosynthesis in safflower floral tissues [36]. Using a prokaryotic expression system, the ability of *CtUGT.144*, which forms a NAT pair with lncRNA *MSTRG.31559* and *CtCGT* to modify flavonoid metabolites was evaluated with kaempferol and luteolin as substrates, respectively. The results suggest that both enzymes possess catalytic activity toward the tested substrates (Figure 5e). *CtCHS.7* and *CtUGT.144*, which are associated with lncNAT pairs, possess catalytic activities related to flavonoid biosynthesis in the flavonoid biosynthesis pathway and exhibit significant differential up-regulation during the flowering stage in the red-flowered safflower variety Yunhong-7, suggesting that lncNAT-associated genes may participate in flavonoid biosynthesis-related processes associated with safflower flower coloration.

## 3. Discussion

Early studies regarded lncRNAs as transcriptional noise due to their low expression levels and sequence conservation. To date, lncRNAs have increasingly gained recognition as key regulatory molecules in both animals and plants. Although lncRNAs were relatively understudied in earlier research, growing evidence now indicates that lncRNAs, together with mRNAs and miRNAs, play a coordinated role in regulating plant growth and development as well as responses to biotic and abiotic stresses [37,38]. Previous studies have indicated that lncRNAs participate in pigmentation and pigment biosynthesis, potentially affecting flower color formation through the regulation of key structural genes. For instance, *TCONS_01039552* and *PONTK.3920.2* were reported to regulate F3H expression in sea buckthorn fruit and potato (*Solanum tuberosum*), respectively [39,40]. However, few studies on lncRNAs in safflower have been reported. In this research, lncRNA sequencing was performed on safflower flowers of different colors. A total of 208 lncNATs were identified as candidate NAT pairs potentially associated with coding gene expression, suggesting that lncNATs may contribute to the development process of safflower flowers and the formation of flower color through direct or indirect mechanisms.

By integrating four lncRNA identification approaches (UniProt, PlantLncBoost, LncFinder-plant, and CPAT-plant), this study identified a total of 4851 lncRNAs. Numerous studies have demonstrated that lncRNAs exhibit widespread tissue-specific expression patterns [41,42]. Pairwise comparisons of lncRNAs across different tissues and developmental stages in strawberry (*Fragaria vesca*) reveal significant differences in their expression levels and genomic distribution [43]. The lncRNAs in safflower exhibit significant variety-specific and developmental stage-specific expression patterns, suggesting their potential involvement in the regulation of characteristic traits across different safflower varieties and developmental stages (Figure 2a,d). Subsequently, by analyzing differential expression patterns between lncRNAs and their corresponding protein-coding genes, a total of 208 lncNATs were identified as potential regulators of coding gene expression. Among them, 114 lncNATs function exclusively during safflower floral development, while 16 and 20 lncNATs specifically regulate variety-specific expression in the flower bud stage and flowering stage, respectively (Figure 3d). The enrichment analysis revealed that lncNATs are involved in the regulation of both flavonoid and carotenoid biosynthesis, indicating that safflower flower color formation may be coordinately influenced by multiple pigments (Figure 3e). The role of carotenoids in safflower flower color formation warrants further investigation in future studies.

The crucial role of lncRNAs in pigmentation has been well established by numerous studies. In the plant of Solanum tuberosum, *PONTK.2668.1* and *PONTK.2668.15* have been shown to regulate the expression of the CHS gene [40]. In this study, the lncRNA *MSTRG.28365* forms a convergent-type NAT pair with *CtCHS.7*, and its expression pattern is inversely correlated with that of *CtCHS.7*, suggesting that MSTRG.28365 may be involved in the regulation of *CtCHS.7* expression during the flowering stage in safflower (Figure 3f,g). WGCNA further assigned *CtCHS.7* to the ME5 module, which was significantly enriched in flavonoid biosynthesis-related functional genes, including the glycosyltransferase *CtUGT.144*, cytochrome P450 hydroxylase *CtCYP76C.2*, and transcription factors from the MYB, bZIP, and NAC families (Figure 4d). The lncRNAs can form NAT pairs with transcription factor, thereby regulating its expression and subsequently influencing anthocyanin biosynthesis and pigment accumulation in flower organs. For instance, reduced expression of MYB1 in Chinese bayberry suppresses anthocyanin biosynthesis in bagged fruits, while the transcriptional level of MYB17 in apples shows a significant positive correlation with anthocyanin accumulation [44]. Meanwhile, *CtUGT.144* and *CtCYP76C.2* also form convergent-type NAT pairs with lncRNAs, indicating that lncRNAs play a broad regulatory role in flavonoid biosynthesis in safflower (Figuer 4e).

Genetic structure analysis of the CHS gene family in safflower revealed that *CtCHS.7* possesses an additional exon relative to other CHS members (Figure 5a). Studies in rice have demonstrated that the fourth exon of *OsMFT1* and *OsMFT2* confers antagonistic functional roles to these two proteins in the regulation of panicle germination [45]. The additional exon of *CtCHS.7* harbors potential lncRNA binding sites, conferring the potential for lncRNA-mediated regulation (Figure 5c). The in vitro enzymatic assay demonstrated that CtCHS.7 catalyzes the formation of a novel product with a maximum absorption wavelength of 398 nm, whose spectral profile is markedly distinct from that of the theoretical product naringenin chalcone (Figure 5d). In *Brunfelsia acuminata* flowers, CHS expression is significantly positively correlated with anthocyanin accumulation [46]. Anthocyanin accumulation is an important determinant of flower color, and the elevated expression of *CtCHS.7* in the Yunhong-7 cultivar may be associated with its red flower phenotype. Although the present study identified multiple candidate lncNAT–coding gene associations through transcriptomic analyses, direct regulatory relationships have not yet been experimentally validated. In particular, the proposed interaction between *MSTRG.28365* and *CtCHS.7* is currently supported primarily by NAT pairing, inverse expression patterns, and co-expression analyses. Future studies involving transient expression assays, RNA interference, CRISPR-mediated perturbation, RNA pull-down experiments, and metabolite quantification will be necessary to clarify the molecular mechanisms underlying lncRNA-associated flower color regulation in safflower.

This study has several limitations. First, the proposed regulatory relationships between lncNATs and coding genes were inferred mainly from transcriptomic correlations and NAT pairing, and direct molecular interactions remain to be experimentally validated. Second, pigment metabolites were not quantitatively measured, limiting the interpretation of the relationship between gene expression and flower coloration. In addition, evolutionary analyses of *CtCHS.7* were not conducted. Future studies integrating metabolomics, transient expression assays, and comparative genomic analyses will help clarify the biological functions of candidate lncNATs in safflower.

## 4. Materials and Methods

### 4.1. Plant Material and Growth Conditions

This study utilized three safflower materials exhibiting different flower colors: Yunhong-7 (red flower), CtWW (white flower), and CtYY (yellow flower), with seeds obtained from the seed resource bank of South-Central Minzu University.

Plants were grown under natural field conditions in a safflower experimental field in Danzhou, Hainan, China. The local photoperiod in January is approximately 11.0–11.5 h light/12.5–13.0 h dark, with light intensity of 300–800 μmol·m^−2^·s^−1^ during sunny mornings. Field conditions included a daytime temperature of 24–28 °C, nighttime temperature of 18–22 °C, and relative humidity of 65–85%. Plants were cultivated in well-drained sandy loam soil and irrigated every 3–5 days based on rainfall and soil moisture, maintaining approximately 60–70% field capacity.

All plant materials (Figure 1a) were collected between 08:00 and 11:00 on 27 January 2024, under sunny conditions to minimize circadian effects. Samples were harvested at the same developmental stage under uniform field management. Immediately after collection, samples were flash-frozen in liquid nitrogen and stored at −80 °C for subsequent RNA extraction. Transcriptome libraries were then prepared for high-throughput sequencing. Three biological replicates were collected for each combination of flower color and developmental stage (3 flower colors × 2 developmental stages × 3 biological replicates), resulting in a total of 18 transcriptome libraries.

Floral developmental stages were defined based on morphological characteristics, with reference to previous studies and adjusted according to the specific features of the experimental materials. The bud stage was defined as the period prior to petal opening, during which the petals remained enclosed by sepals and showed varying degrees of pigment accumulation. The full bloom stage was defined as the stage at which petals are fully expanded and tubular florets are clearly visible, representing a mature stage with stable flower coloration. All samples were collected at comparable physiological states to minimize biological variation (Figure 1b).

### 4.2. Strand-Specific cDNA Library Preparation and ssRNA-Seq

Total RNA was extracted from the flower tissues of three safflower materials at the bud stage and the flowering stage. Subsequently, mRNA was enriched from the total RNA using oligo(dT) beads. After fragmentation, the fragmented mRNA was reverse-transcribed into first-strand cDNA using random primers, followed by second-strand cDNA synthesis along with dUTP substituted for dTTP. Then, the double-stranded cDNA was subjected to end repair, A-tailing, and adapter ligation. Finally, PCR amplification was performed to construct strand-specific sequencing libraries. Strand-specific RNA sequencing (ssRNA-seq) was conducted on the DNBSEQ platform.

RNA integrity was initially evaluated by 1% agarose gel electrophoresis to assess potential degradation and genomic DNA contamination. RNA purity was measured using a NanoDrop One UV-Vis spectrophotometer (Thermo Fisher Scientific, Waltham, MA, USA), with acceptable absorbance ratios of A260/A280 ranging from 2.0 to 2.2 and A260/A230 ranging from 1.8 to 2.1. RNA concentration was accurately quantified using a Qubit fluorometer (Thermo Fisher Scientific, Waltham, MA, USA), and RNA quality was further assessed with an Agilent 2100 Bioanalyzer (Agilent Technologies, Santa Clara, CA, USA). Only samples with RNA integrity number (RIN) values ≥ 8.0 and 28S/18S ratios ≥ 1.5 were used for downstream analyses.

First-strand cDNA was synthesized from total RNA using the HiScript II 1st Strand cDNA Synthesis Kit (Vazyme, Nanjing, China) according to the manufacturer’s instructions, and the resulting cDNA samples were stored at −20 °C until use. To validate transcriptome sequencing (RNA-seq) results, four differentially expressed genes (*CtYH8G243210*, *CtYH10G318070*, *CtYH11G331460*, *CtYH8G220570*) were selected for quantitative real-time PCR (qPCR) analysis. qPCR reactions were performed in triplicate using the ChamQ Universal SYBR qPCR Master Mix on a QuantStudio 7 Flex Real-Time PCR System (Thermo Fisher Scientific, Waltham, MA, USA). Gene expression levels were calculated using the 2^−ΔΔCt^ method and normalized to the internal reference gene Actin. Primer sequences are listed in Supplementary Table S1.

### 4.3. Transcript Assembly and lncRNA Identification

Paired-end (2 × 150 bp) strand-specific sequencing reads from each sample were aligned to the Yunhong-7 reference genome with HISAT2 (v2.2.1) [47]. Transcript assemblies were reconstructed from the alignments by StringTie (v2.1.7) [48], and all individual assemblies were merged into a non-redundant transcript set using the “stringtie--merge” program. After removing transcripts shorter than 200 bp, the retained sequences were aligned to the UniProt database to exclude transcripts with protein-coding potential. PlantLncBoost [49], LncFinder-plant [50], and CPAT-plant [51] were subsequently applied to identify lncRNAs from the filtered transcripts. Only transcripts consistently identified as lncRNAs by all three tools were retained for further analysis.

lncRNAs overlapping protein-coding gene regions were identified using BEDTools [52]. lncRNAs and protein-coding genes located on opposite strands within overlapping genomic regions were classified as natural antisense transcript (NAT) pairs.

### 4.4. Analysis of lncRNA and mRNA Expression

Strand-specific RNA-seq reads were first mapped to the Yunhong-7 reference genome using HISAT2 (v2.2.1). Transcript abundance for both protein-coding genes and lncRNAs was then estimated with StringTie (v2.1.7). Protein-coding gene annotations were obtained from our previously assembled Yunhong-7 genome, whereas lncRNAs were annotated based on the pipeline described above.

To identify differentially expressed transcripts among safflower materials at different flower developmental stages, we employed DESeq2 (v1.38.3) in R. Transcripts with an FDR ≤ 0.05 and |log2 fold change| ≥ 1 were regarded as differentially expressed. Expression patterns were visualized as heatmaps using the pheatmap package (v1.0.13).

TPM (transcripts per million) data of differentially expressed genes from 18 samples were used as input data, and the weighted gene co-expression network analysis (WGCNA) package in R (version 4.5.1) was employed to identify highly correlated gene modules [53]. The co-expression network was visualized using Cytoscape software (version 3.10.4).

### 4.5. Prokaryotic Expression in E. coli

The full-length coding sequences of *CtCHS.7*, *CtUGT.144*, and *CtCGT* were obtained via PCR amplification from cDNA derived from safflower (Yunhong-7) tubular florets at two developmental stages: the flower bud stage and flowering stage. These sequences were subsequently subjected to homologous recombination with the pET28a vector, which was double-digested with *BamH*I and *Hind*III, followed by transformation into *E. coli* DH5α competent cells. Positive clones were screened on solid Luria–Bertani (LB) medium plates containing 50 μg/mL kanamycin. The plasmids with correct sequencing were re-transformed into *E. coli* BL21 (DE3) competent cells. The seed liquid was inoculated into 300 mL of liquid LB medium (containing 50 μg/mL kanamycin) at a ratio of 1% (*v*/*v*), and cultured at 37 °C and 200 rpm until OD600 reached 0.6–0.8. IPTG was added to a final concentration of 400 μM, and the culture was continued at 16 °C and 200 rpm for 20 h. The cells were collected by centrifugation, resuspended in pre-cooled Buffer A, and disrupted using a high-pressure homogenizer. The lysate was centrifuged at high speed for 30 min, and the supernatant was filtered through a 0.22 μm filter and purified using a protein purification instrument and a His Trap FF 5 mL column (Cytiva, Marlborough, MA, USA).

Enzyme activity was determined in a 100 μL system (50 mM pH 7.5 phosphate buffer, 10 mM kaempferol, 10 mM luteolin, 100 μg recombinant protein) at 37 °C overnight.

Kaempferol and luteolin stock solutions (10 mM) were added at 2 μL each. The reaction was terminated by adding two volumes of acetonitrile. The reaction mixture was centrifuged at 12,000 rpm for 10 min, and the supernatant was analyzed by HPLC: YMC-Triart C18 column (4.6 mm × 250 mm, 5 μm), 25 °C, 1.0 mL/min, with the mobile phase A (water + 0.1% formic acid) and B (acetonitrile) eluting in a gradient of 0–20 min 5 → 100% B, 20.1–25 min 100 %B, 25.1–28 min 5% B.

Buffer A consisted of 50 mM Tris-HCl, 300 mM NaCl, 20 mM imidazole, and 10% (*v*/*v*) glycerol (pH 8.0). Buffer B consisted of 50 mM Tris-HCl, 500 mM NaCl, 250 mM imidazole, and 10% (*v*/*v*) glycerol (pH 8.0). The storage buffer contained 100 mM sodium dihydrogen phosphate dodecahydrate, 100 mM disodium hydrogen phosphate dihydrate, and 10% (*v*/*v*) glycerol (pH 7.4). All buffers were filtered prior to use.

Prokaryotic Expression Material: The *E. coli* strain DH5α used for prokaryotic expression is maintained in our laboratory.

## 5. Conclusions

This study identified a total of 4851 lncRNAs using strand-specific sequencing, among which 940 were found to form natural antisense transcript (NAT) pairs with protein-coding genes. Of these, 208 lncNATs were predicted to potentially regulate gene expression. Functional enrichment analysis indicated that these lncRNAs were mainly associated with pathways involved in pigment biosynthesis and regulation, such as flavonoid biosynthesis, carotenoid biosynthesis, and plant hormone signal transduction, implying a possible role in flower color formation.

Several key genes related to flavonoid biosynthesis, including *CtCHS.7*, *CtUGT.144*, and *CtCYP76C.2*, were identified as candidate genes potentially associated with lncRNA-mediated regulation. Among them, *CtCHS.7* appears particularly noteworthy. Compared with typical CHS genes, *CtCHS.7* contains additional exons, which may increase its regulatory complexity and provide more potential sites for interaction with lncRNAs. This structural feature suggests that *CtCHS.7* may represent a candidate target potentially associated with lncRNA-mediated regulation at both the transcriptional and post-transcriptional levels.

Previous studies have shown that members of the chalcone synthase (CHS) superfamily can exhibit functional diversity. In some cases, canonical CHS enzymes produce p-coumaroyltriacetic acid lactone (CTAL) as a by-product during polyketide chain elongation, and small changes in active-site residues are sufficient to alter product specificity. In addition, CTAL synthase (CTAS), a CHS-related enzyme, shares high sequence similarity with CHS but shows reduced cyclization efficiency, resulting in the accumulation of CTAL-type compounds.

In this context, *CtCHS.7* may represent a functionally divergent CHS-like enzyme. It is possible that, in addition to being regulated by lncRNAs, *CtCHS.7* also exhibits altered catalytic properties, favoring chain elongation over cyclization. Such a combination of potential regulatory association and enzymatic variation may contribute to the diversification of flavonoid metabolites in safflower.

Overall, these results expand our understanding of the regulatory framework underlying flavonoid biosynthesis and provide a basis for future studies on the molecular mechanisms of flower color formation in safflower.

## Data Availability

The strand-specific RNA-seq data for three safflower varieties have been deposited in the National Genomics Data Center (https://ngdc.cncb.ac.cn/) under the accession PRJCA054695.

## Supplementary Materials

The following supporting information can be downloaded at: https://www.mdpi.com/article/10.3390/ijms27115142/s1.
