# Supplementary material for: Transcriptome Dynamics Reveal the Potential Roles of Long Non-Coding RNAs in Regulating Flower Color of Safflowers (Carthamus tinctorius)
Source: Int J Mol Sci. 2026 Jun 5;27(11):5142. doi: 10.3390/ijms27115142 (PMC13257136; doi:10.3390/ijms27115142)

Supplementary Figure S1. The transcriptome expression profiles of the candidate genes, together with their qPCR-validated expression levels, were examined in three cultivars (Yunhong-7, CtYY, and CtWW) at the flower budding and full bloom stages.

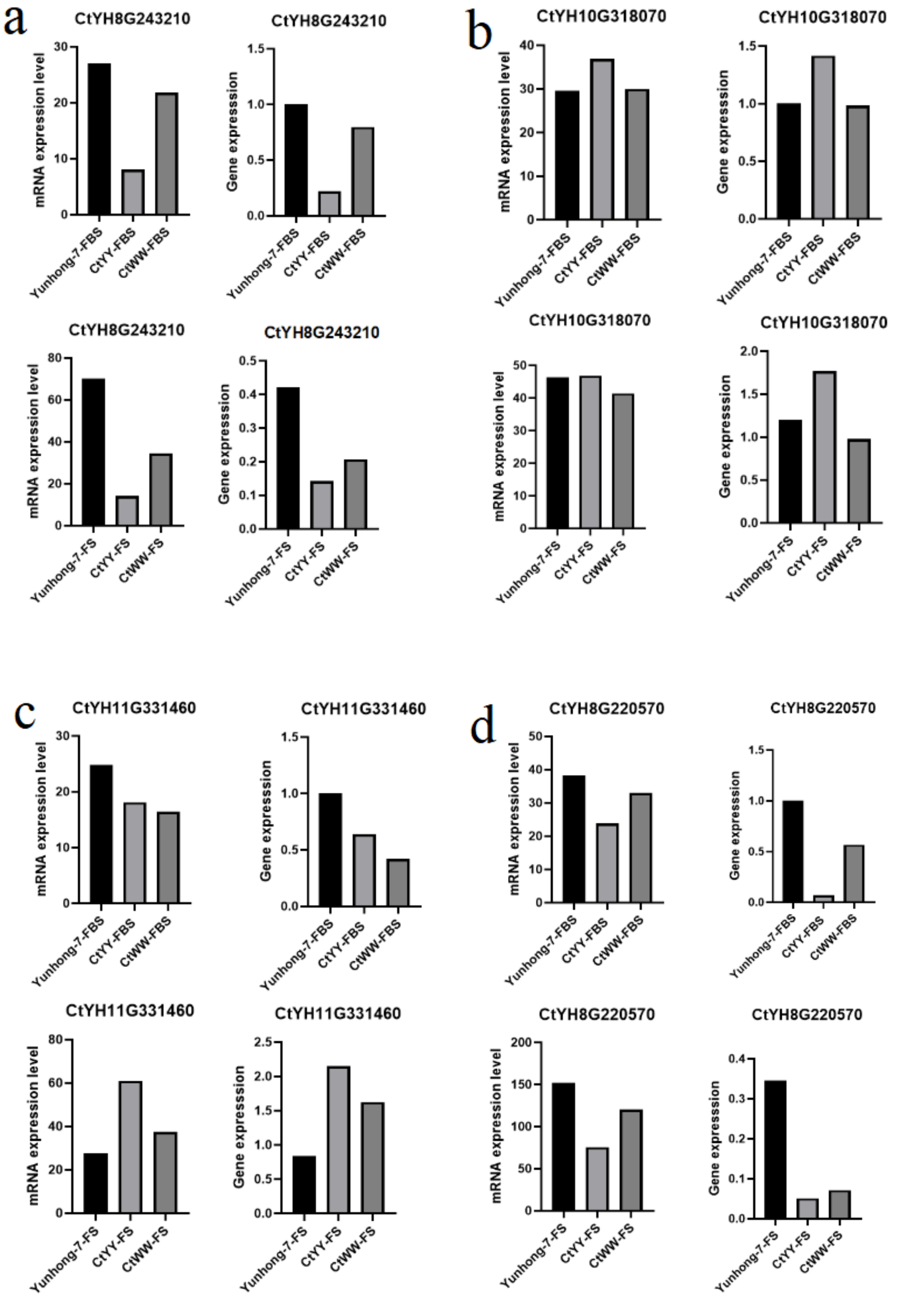

Supplementary Figure S2. Pearson correlation coefficient of 18 strand-specific RNA-seq (ssRNA-seq) datasets. Gene expression levels (estimated as TPM [Transcripts Per Million] values) of lncRNAs were used for this analysis. Three replicates were performed for each accession. CtWW, CtYY and Yunhong-7 indicate white, yellow and red flower of *Carthamus tinctorius*, respectively. FBS and FS represent flower bud stage and flower stage, respectively.

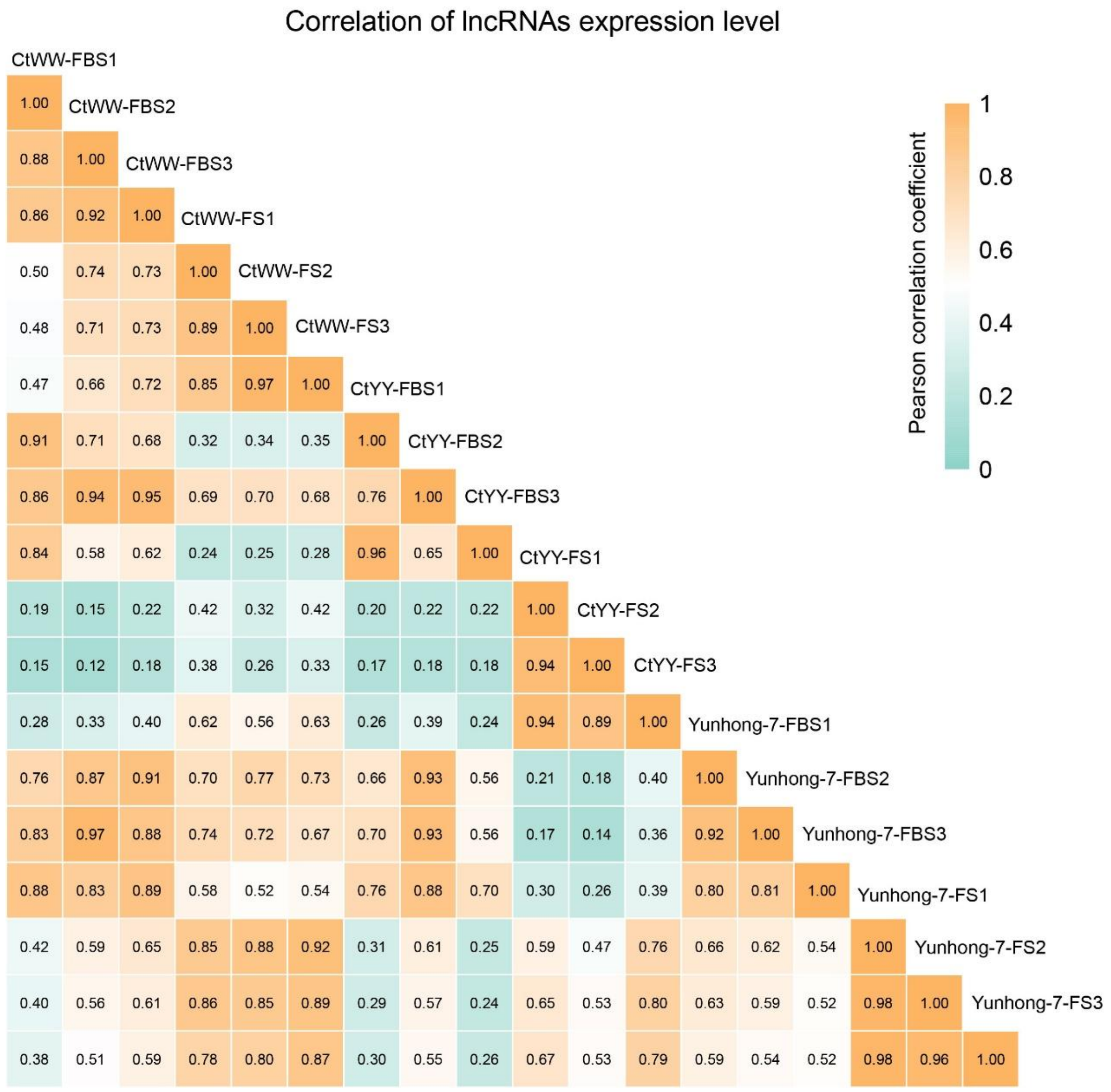

Supplementary Figure S3. Pearson correlation coefficient of 18 strand-specific RNA-seq (ssRNA-seq) datasets. Gene expression levels (estimated as TPM [Transcripts Per Million] values) of mRNAs were used for this analysis. Three replicates were performed for each accession. CtWW, CtYY and Yunhong-7 indicate white, yellow and red flower of *Carthamus tinctorius*, respectively. FBS and FS represent flower bud stage and flower stage, respectively.

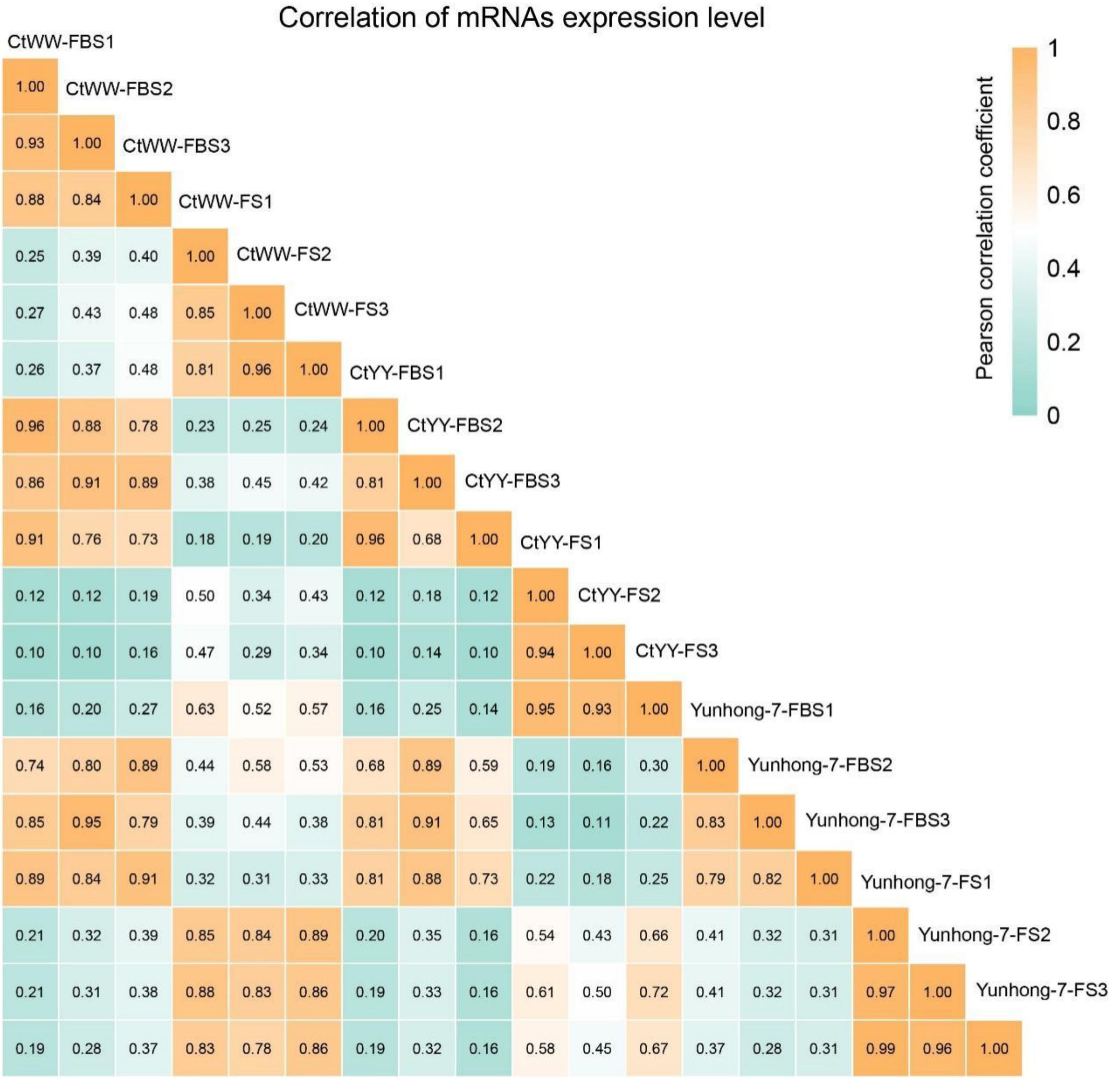

Supplementary Figure S4. Expression levels (log2FPKM) of different RNAs in Yunhong-7, CtWW and CtYY under flower bud stage (FBS) and flower stage (FS).

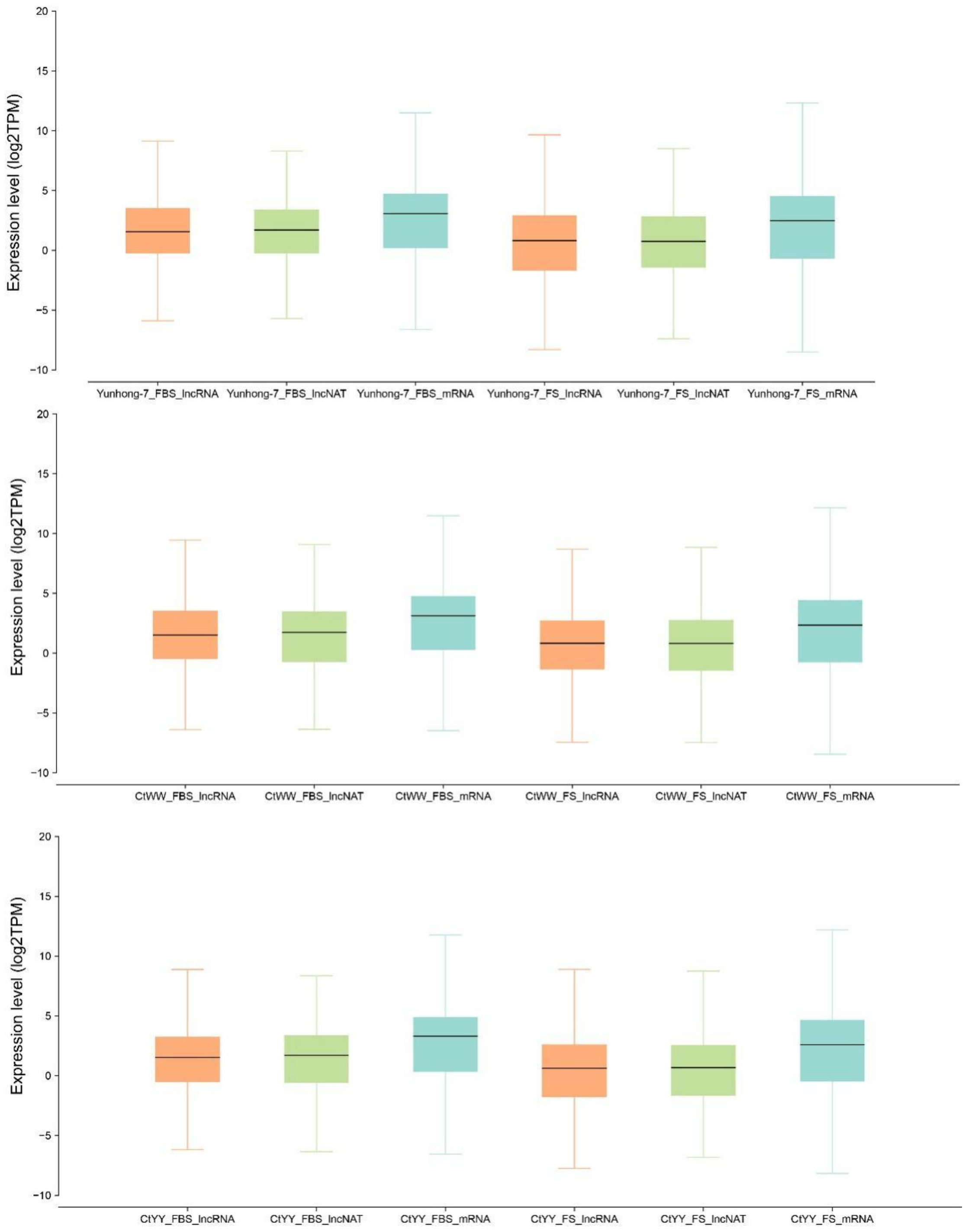

Supplementary Figure S5. KEGG enrichment analysis of coding genes from NAT pairs.

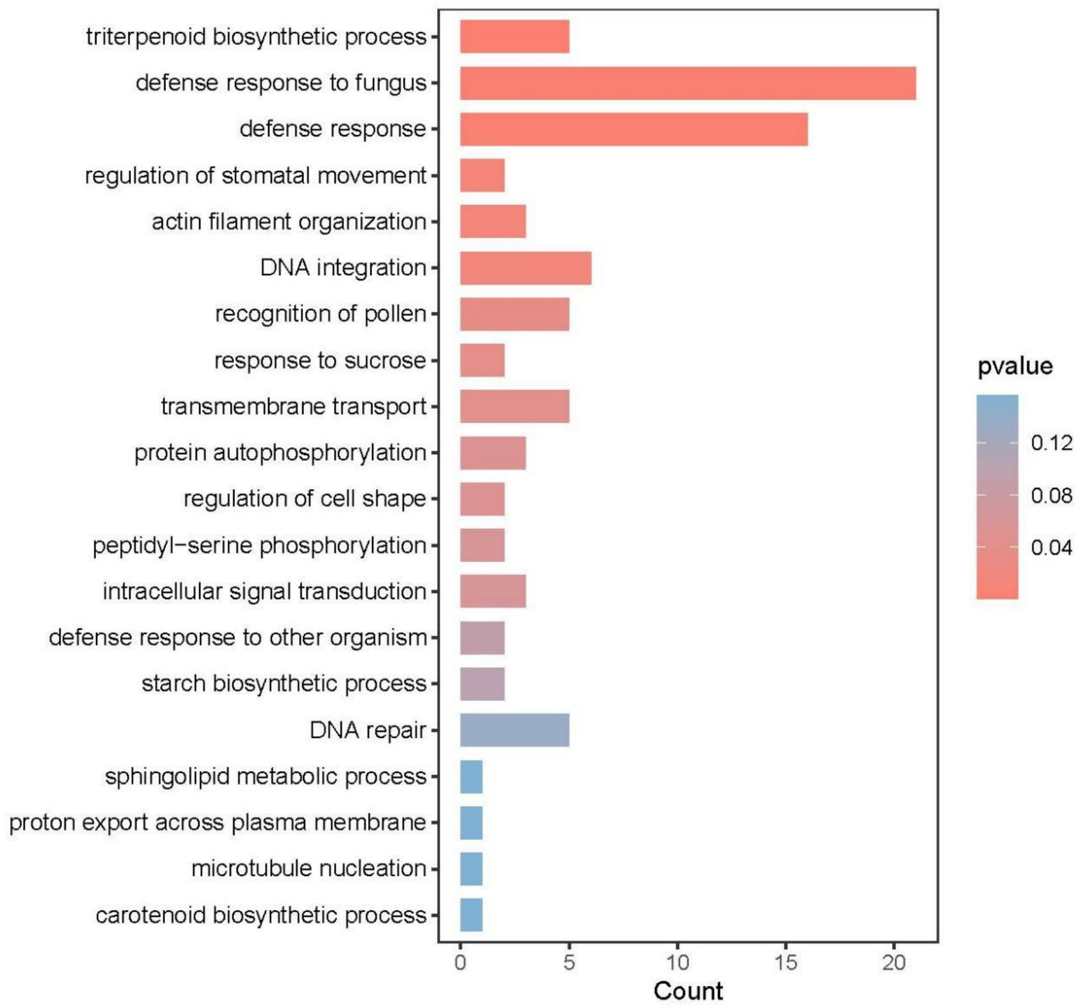

Supplementary Figure S6. Venn diagram of differentially expressed genes across three safflower varieties.

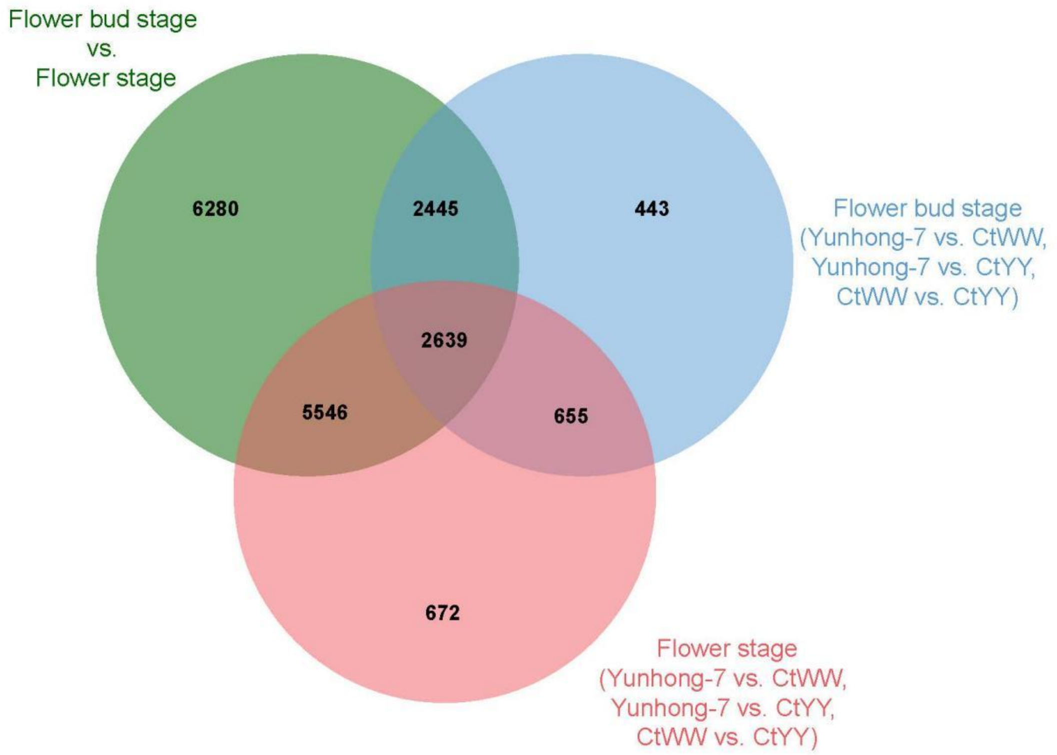

Supplementary Figure S7. Venn diagram of differentially expressed lncRNAs across three safflower varieties.

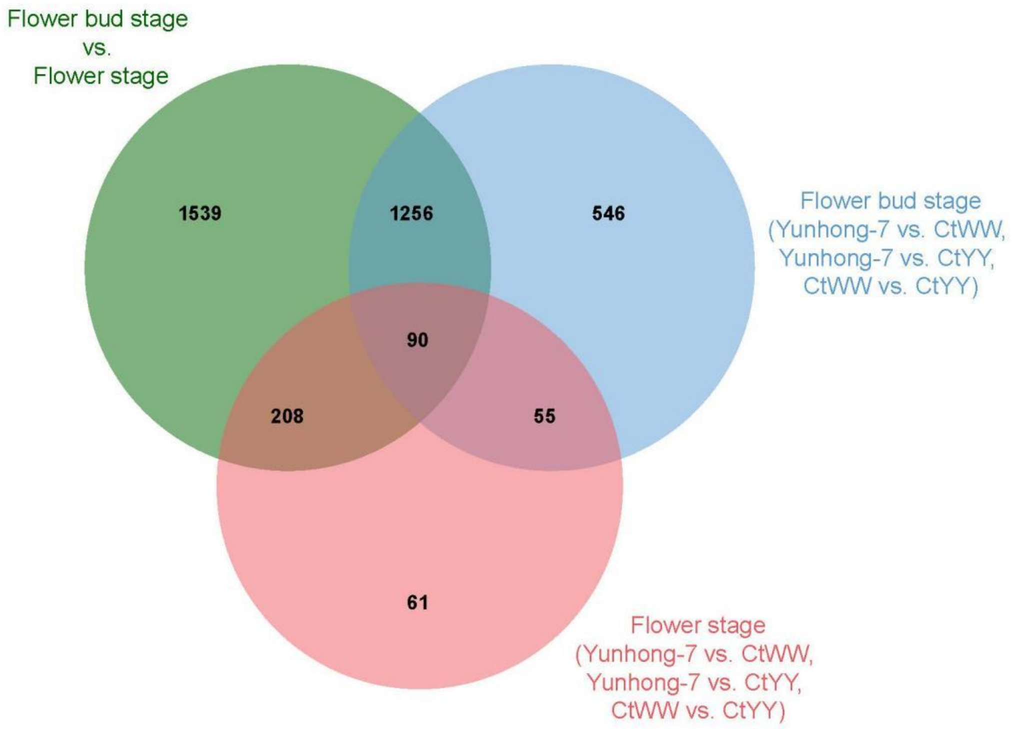

Supplement: Supplementary file 1 [file ijms-27-05142-s001.zip › Supplementary Figures.pdf]
